# Hip–Knee Coupling Exoskeleton With Offset Theory for Walking Assistance

**DOI:** 10.3389/fbioe.2021.798496

**Published:** 2022-01-31

**Authors:** Jianfeng Ma, Decheng Sun, Xiao Chen

**Affiliations:** ^1^ Department of Materials and Manufacturing, Beijing University of Technology, Beijing, China; ^2^ The Quartermaster Engineering and Technology Research Institute, Institute of System Engineering, AMA., PLA, Beijing, China

**Keywords:** exoskeleton, couple mechanism, offset theory, joint power consumption, walking assistance

## Abstract

To alleviate the influence of aging on the elderly, a hip–knee coupling exoskeleton with offset theory is designed in this article to improve people’s athletic ability. With the novel design, the unique application strategy of the offset principle for hip and knee joints is developed, and a hip–knee coupling mechanism is proposed to solve the discrete power assistance problem for the knee joint. To acquire the success of the design, the mathematical model of the coupling mechanism is established to optimize the load environment of the exoskeleton system, and furthermore, an algorithm adapted to human movement is proposed to determine the monotonicity of the cam profiles. For the selection of the elastic parameters in the coupling mechanism, the sensitivity condition is proposed, and the human–machine interaction model of the KESM is further established. A man–machine coupling model was used to verify the scientificity of the exoskeleton design, and the comparison between the joint powers with or without exoskeleton indicated that the exoskeleton theoretically saved at least 20% of the human body’s energy.

## 1 Introduction

When a person gets older, the deceleration of muscle strength and joint function brings much confusion to the daily activities of the elderly (Brown et al., 2020). This article intends to alleviate such problems from the direction of mechanical engineering, hoping to design a dexterous mechanism that provides walking assistance to humans. The lower limb exoskeleton provides a new design perspective, which is advantageous, compared with other walking aids, as it can accompany the human into various environments.

The lower limb exoskeleton can be divided into powered exoskeleton and passive exoskeleton according to different functional principles (Schick et al., 2020). The powered exoskeleton directly provides external energy to human motion, in which the actuators are used to convert hydraulic energy, electrical energy, or pneumatic energy into mechanical energy required by the human body (Stauffer et al., 2009; Talaty et al., 2013; Tsukahara et al., 2015; Lovrenovic and Doumit, 2016; Li et al., 2017; Munera et al., 2017; Ding et al., 2018; Niu et al., 2018; Pan et al., 2018; Zhang et al., 2018; Chen et al., 2019; Schick et al., 2020). The powered exoskeleton has made remarkable achievements in human walking assistance (Pardoel and Doumit, 2019). However, the ambiguity and complexity during the intention recognition for human motion is a dangerous potential factor for the elderly with poor balance.

Passive exoskeleton has more advantages in compactness than powered exoskeleton (Lovrenovic and Doumit, 2016). The essential function of passive exoskeleton is to allocate energy consumption more reasonably in human motion because the exoskeleton does not provide additional energy. The way of energy redistribution depends on the structural design. Different structural designs have different meanings for human motion.

Reducing the influence of gravity on the human body is a method of exoskeleton design. Passive exoskeleton with gravity compensation function can reduce the muscle strength in patients suffering from muscle weakness (Nathan, 1985; Agrawal and Fattah, 2004; Banala et al., 2006; Fattah et al., 2006; Rahman et al., 2007; Koser, 2009; Nakayama et al., 2009; Arakelian, 2016; Hung et al., 2017; Alamdari et al., 2019; Zhou et al., 2020). In the assumed conservation process for the mechanical energy of the human–machine system, the joint torque that represents the energy consumption of the human body is an important factor that is ignored. The devices (Lee and Wang, 2015; Lovrenovic and Doumit, 2019) fulfilled its power assistance function by taking advantage of changes in gravitational potential energy as the body walks, in which the exoskeleton gives the pelvis an upward force to reduce the load of the user’s knee joint. However, the wearer’s kinematics tests show that the exoskeleton interferes with the human body.

The mutual transformation between the joint positive and negative work is another way to realize the power assistance function of the exoskeleton. The ankle exoskeleton is a small exoskeleton structure around the ankle joint, which has an elastic component parallel to the calf muscle to reduce its metabolic energy (Wiggin et al., 2011; Collins et al., 2015; Yandell et al., 2019). References (van den Bogert, 2003; van Dijk et al., 2011; van Dijk and Van der Kooij, 2014) propose a design idea of the artificial tendon to reduce muscle strength, which realizes the conversion between positive and negative work in multiple joints. However, the construction of the system is complicated. Specifically, the test results for the prototype (van Dijk and Van der Kooij, 2014) are far less than the expected benefits.

Based on the development of exoskeletons, an easy-to-implement theoretical method for the hip and knee joints is proposed in this article, which is called the offset principle. Its essence is to realize the mutual transformation between the joint positive and negative work in a single joint, in which the unique application strategy based on human mechanics is presented. Specifically, to adapt to the variable stiffness characteristics of the knee joint, a hip–knee coupling mechanism based on multi-joint linkage is proposed in this article to solve the discrete power assistance problem for the knee joint. This article provides a set of theoretical references for the design of the hip–knee coupling mechanism, which includes motion planning, system modeling, optimization algorithms, and sensitive conditions.

Specifically, chapter 3 covers the design principle and structure design of the exoskeleton. In particular, the motion process of the coupling mechanism is predicted. In chapter 4, a mathematical model is developed to optimize the load condition of the coupling mechanism. In particular, an algorithm is proposed to determine the monotonicity of the cam profiles. Chapter 5 is applied to the parameter design of elastic elements in the coupling mechanism. In particular, the sensitivity condition is proposed, and furthermore, the human–machine interaction model for the knee energy storage mechanism is established. In chapter 6, a design index for the effectiveness of the exoskeleton based on the offset theory is proposed, and the power assistance effect of the lower limb exoskeleton on the human body is proven.

## 2 The Exoskeleton Design Based on the Offset Theory

### 2.1 The Content of the Offset Theory

The exoskeleton design is based on the offset theory, the mutual offset between joint positive and negative work that includes the unique implementation strategies for the hip and knee joint. Specifically, the judgment method of positive torque is that the direction of the joint torque is the same as the vector direction of joint rotation. The functional principle of the exoskeleton is similar to the tendon structure in the biological motion system—through the passive elastic structure, the energy consumption at the joints is more rationally distributed throughout the gait cycle.

Specifically, for the hip energy storage mechanism (the HESM), there are two conversion nodes (node A and node B) between positive and negative works during the whole gait cycle (Figure 2). The ideal conversion is to store the negative work between node A and node B, and transfer it to the positive work in the region after node B. However, it must be clear to grasp the position of node A; otherwise, it will cause an additional burden to the human body in the part of the time before node A. The position of node A varies from person to person, which is an unknown factor that cannot be clearly defined. However, taking into account the energy conservation law, the energy absorbed from the human body will eventually be compensated for the movement of the lower limbs, which can provide more energy for the human body in the power assistance period. Therefore, this article chooses the period to store energy when the hip joint swings backward and feedback to the human body when the hip swings forward.

**FIGURE 1:**
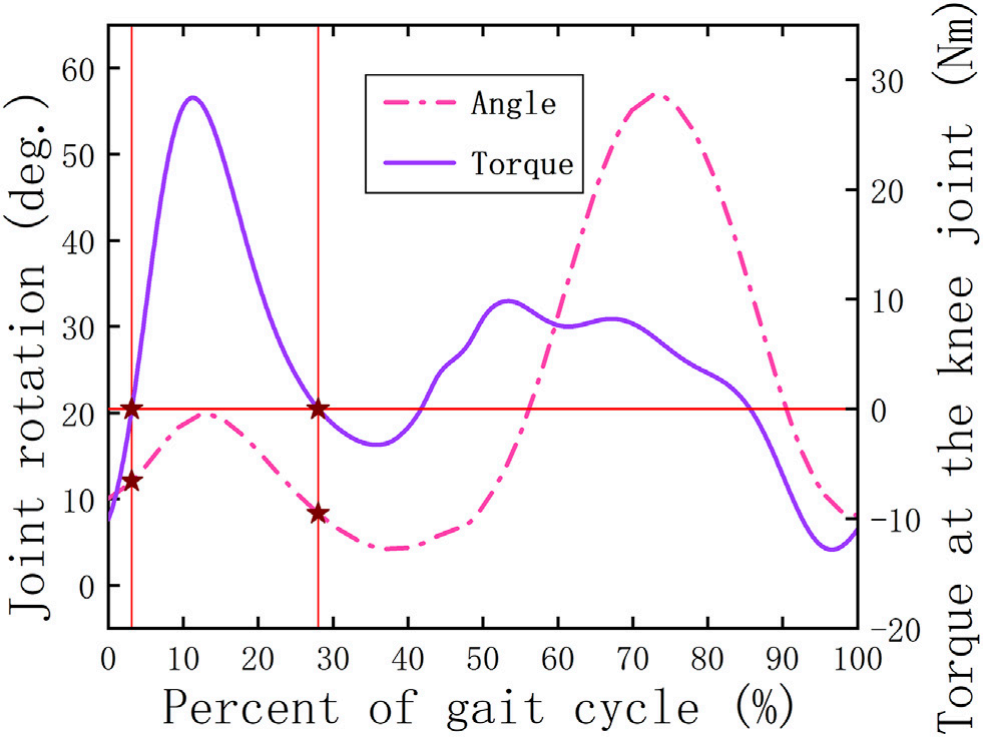
Relationship between the joint torque and angle for the knee joint.

**FIGURE 2:**
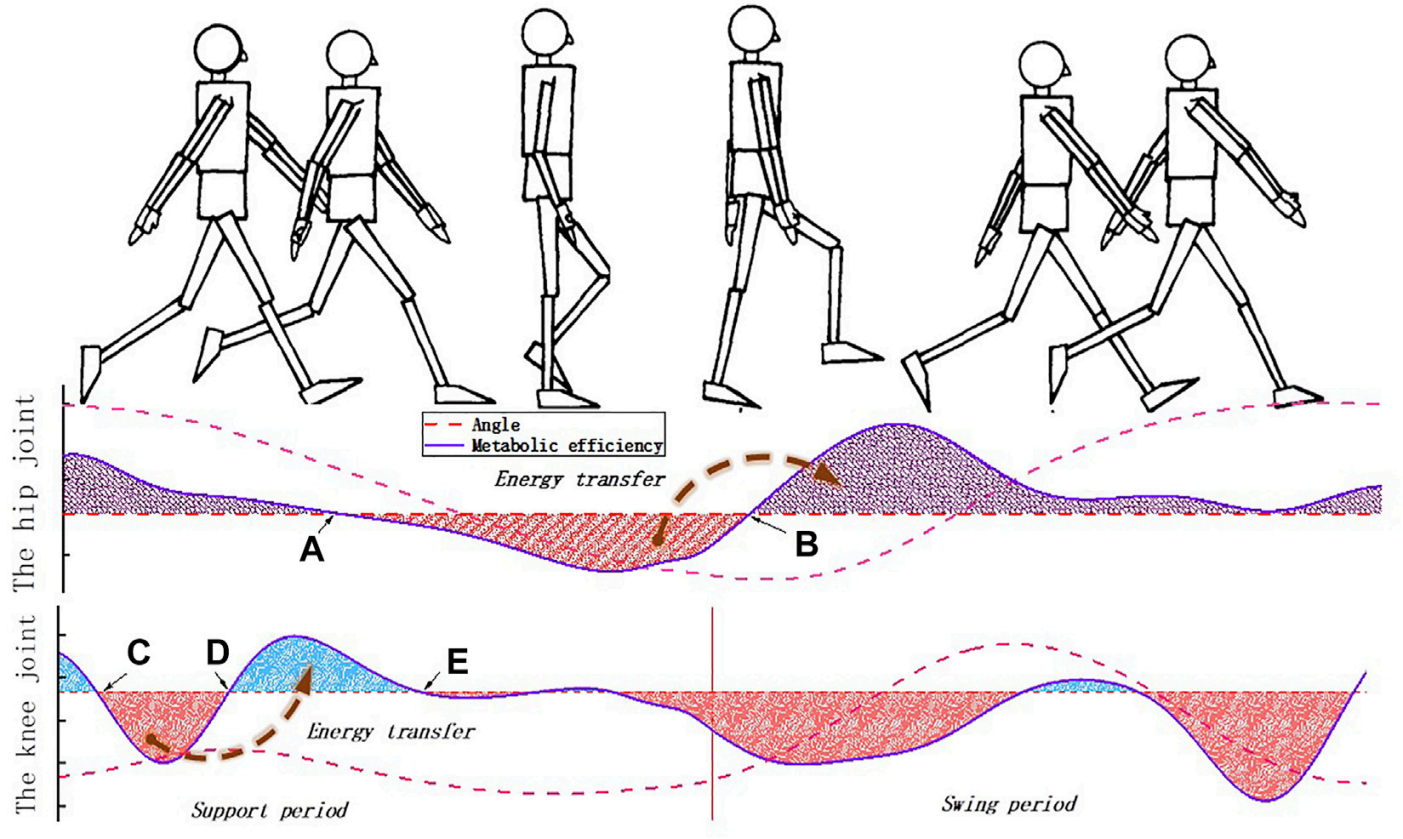
Movement characteristics of the lower limbs of the human body.

Compared with the HESM, the knee energy storage mechanism (the KESM) also needs to solve a discrete power assistance problem. The main responsibility of the knee joint in the swing period is to ensure that the lower limbs complete the initial preparation for the next gait. In this process, in addition to the small amount of energy consumption required for the leg to complete movement, it does not bear other loads (Figure 1); that is, the main task of the knee joint in the swing period is to complete the relevant movement rather than bearing the load. Therefore, the design criterion of the KESM in the swing period is to ensure that there is no kind of obvious interference for the knee joint, including movement and geometry. During the stance period, similar to the hip joint, there is a transition node (the node D) in Figure 2. For the same reasons, the interval between point C and point E is set as the working area of the HESM.

### 2.2 The Structure Design of the Exoskeleton

**FIGURE 3:**
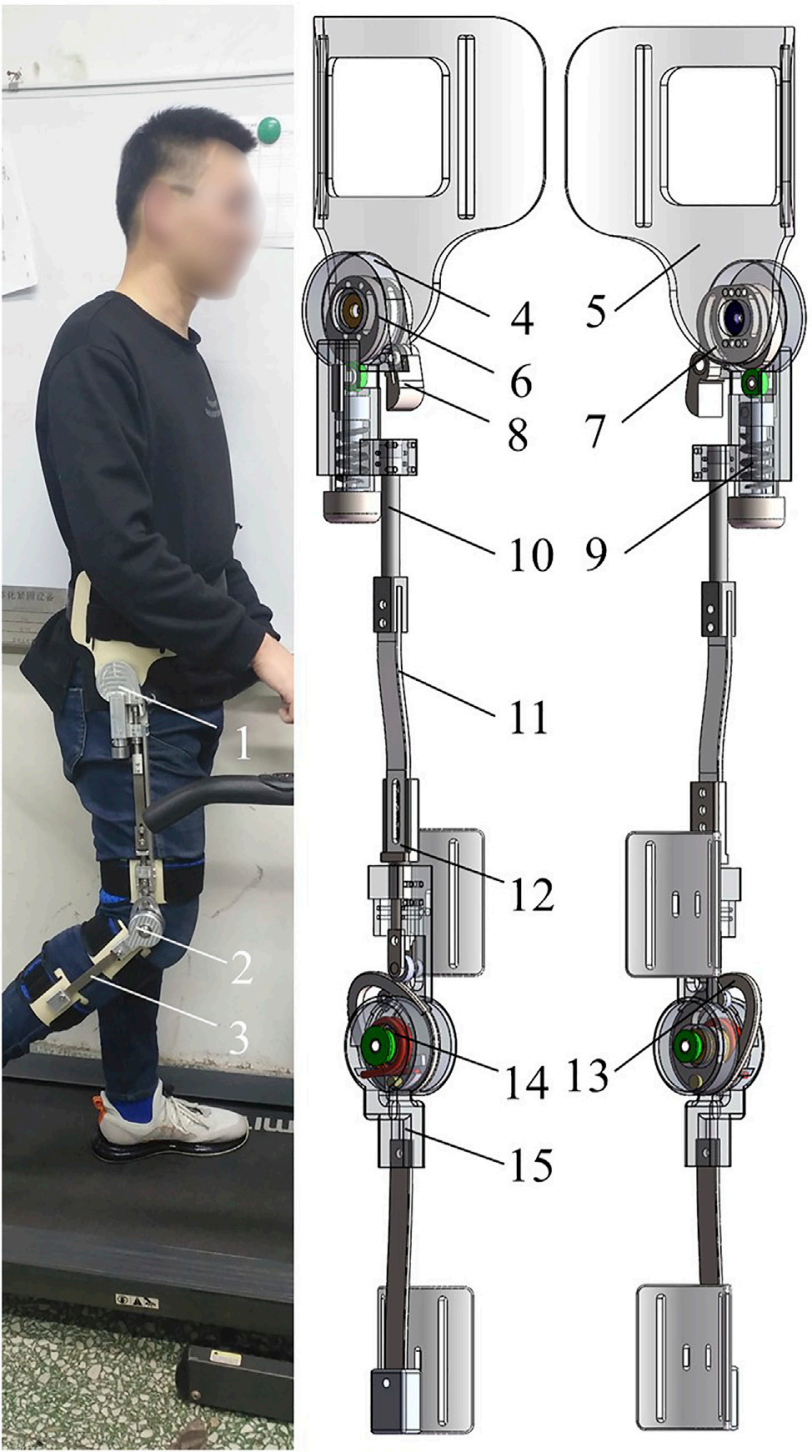
**(A)** human-computer interaction of the exoskeleton. **(B)** Structural design of the exoskeleton. 1-exoskeleton hip, 2-exoskeleton knee, 3-exoskeleton lower leg, 4-hip end cap, 5-waist connection plate, 6-hip master cam, 7-hip deputy cam, 8-pawl, 9-hip spring, 10-pushrod 3, 11-pushrod 2, 12-pushrod 1, 13-knee cam, 14-knee torsion spring, and 15-knee outer cap.

The hip–knee coupling exoskeleton is divided into bilateral structures on both sides, and the basic design principles are consistent. To show its spatial structure as comprehensively as possible, this article takes the right structure as an example to introduce the lower limb exoskeleton (Figure 3). The hip and knee joints of the lower limb exoskeleton are connected by three pushrods (the roller follower) connected by bolts. The design for the roller follower is to form the transfer component of the coupling mechanism and establish a human–machine interaction bridge for the HESM. Each mechanism (the HESM and the KESM) has a personalized knob to adjust the power assistance degree for different populations.

#### 2.2.1 The Design of the HESM

The HESM with one-DoF is used to equivalent hip movement in the sagittal plane (Figure 3A). The hip cam is connected to the waist plate through two centrally symmetric holes in four holes, the purpose of which is to coordinate the hip cam with the pawl at different curvatures, and adjust the relative sliding between the cam and the pawl by changing the extrusion force to ensure that the pawl can move in time. The hip cam is divided into the hip master cam and the hip deputy cam. The hip master cam converts the stroke of the cam mechanism into the deformation of the spring to realize the energy transfer between humans and machines.

The hip master cam, when the hip joint rotates backward, compresses the hip spring to recover the joint negative work, while the hip deputy cam controls the position and orientation of the pawl by rolling extrusion, and further limits the movement of the roller follower during the stance period. The pawl is built-in a torsion spring to ensure that it can be reset in time at the beginning of the swing period. Compared to meshing motion, the reason for using the rolling friction is that there is a complete set of actuating travel and return travel of the cam mechanism in the whole gait cycle. The friction generated by rolling extrusion can fully realize the control of the position and orientation of the pawl in the actuating travel of the hip cam, and the adverse friction can avoid the influence on the position and orientation of the pawl in the return travel of the hip cam.

#### 2.2.2 The Design of the KESM

The KESM is used to equivalent the knee movement in the sagittal plane (Figure 3B) (Xing, 2013). The knee inner cap and the knee outer cap are bound to the human thigh and the human calf, respectively. The two ends of the knee torsion spring are fixed on the knee cam and the knee outer cap, respectively. The knee cam is used to realize the energy storage control of the knee torsion spring. When the pawl completes the limit of the knee cam mechanism during the stance period, the knee cam, the roller follower, and the pawl rest on the human thigh. At the same time, driven by the human calf, the subsequent flexion of the knee joint will be reflected in the deformation of the knee torsion spring, during which the mechanism realized, at the knee joint, the conversion between the joint positive and negative works.

#### 2.2.3 The Motion Law of the Coupling Mechanism

The orderly and periodic joint coupling guarantees normal human movement. Following this orderly movement idea, this article explores the movement rules between the hip and knee joints, and designs a hip–knee coupling mechanism. The essential function of the coupling mechanism is to use the inherent movement habits of the human body to establish the internal coupling law of the exoskeleton, which not only solves the clutch problem in the exoskeleton but more importantly ensures the comfort of the human body in the man–machine interaction. The coupling mechanism is the collection of the hip auxiliary cam, pawl, roller follower, and knee cam, whose function is to transform the motion signal of the hip joint into the on–off signal of the KESM. As a bridge of coupling or interaction between joints, the coupling mechanism is of decisive significance to the realization of the function of the KESM. Its outstanding design features are as follows (Figure 12):

1) The pawl can limit the movement stroke of the roller follower in advance during the support period, which is guaranteed by the timely movement of the pawl that is driven by the rolling extrusion of the hip deputy cam.
2) Under the limit of the claw, the roller follower does not move. The deformation of the knee torsion spring will be influenced by the flexion of the knee joint.
3) During the swinging period, the roller follower’s free movement is not restricted by the pawl.

The current mechanism adjustment scheme is to use the length of the roller follower to adjust the trigger cycle of the coupling mechanism and the fixed position of the cam relative to the waist plate to adjust to the degree of rolling friction adapt to the movement habit.

## 3 The Loading Optimization for the Coupling Mechanism

The coupling mechanism is an important part of the KESM. Its function is to convert the motion law of the hip joint into the clutch signal of the KESM, in which when the roller follower restricts the movement of the knee cam under the movement constraint of the pawl, the rotation of the knee joint will be reflected in the elastic deformation of the knee torsion spring, and further realize the offset between the joint positive and negative works. Therefore, the KESM performs its assist function without relative internal movement and the realization of the restraint relationship of the pawl on the roller follower needs to ensure the stroke amount of the roller follower within the design rotation angle (Figure 12). However, the known quantities at the two points on the cam cannot determine the outer contour of the knee cam, which requires the contour equation to be solved according to the predetermined function requirements in the entire range of motion.

The optimal design of the contour equation of the knee joint cam is necessary. Although the knee cam and the roller follower maintain a static relationship under the pawl constraint, and the contact point between them is constant under the constraints of the rotation angle and the motion stroke, and the influence of other variables on the cam profile will be reflected in the moment arm between the cam and the roller follower at the contact point. When the energy storage of the knee torsion spring reaches its maximum, the size of the moment arm will affect the contact force between the cam and the roller follower, and ultimately affect the load environment of the pawl. The pawl is a force-bearing body with an abrupt cross section, and a good force-bearing condition has a positive meaning for the strength of the exoskeleton.

**FIGURE 4:**
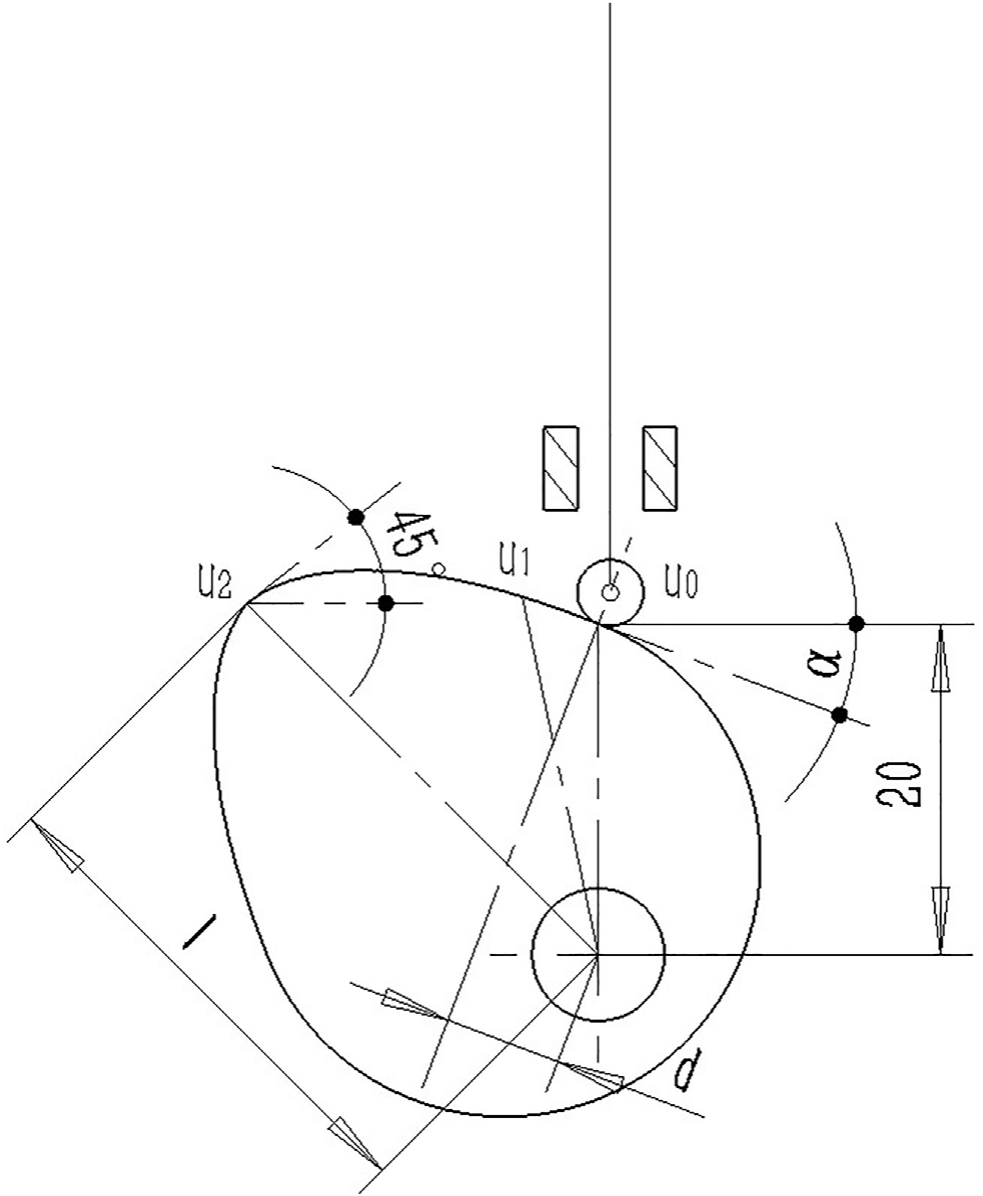
Structure diagram of the knee cam and roller follower.

The schematic diagram of the knee cam and roller follower is shown in Figure 4, in which, to obtain a compact design, the cam is designed to be symmetric about the −45° axis to reduce the stroke of the roller follower.

To ensure that the internal motion of the coupling mechanism meets the design objectives of this article, based on previous design experience, taking into account the structural load, structural size and continuity of internal motion, and other factors, the basic design requirements for the knee cam are shown in Table 1. As shown, α and l are the unknown design quantities that need to be selected according to the change of the moment arm of the contact point.

**TABLE 1:** Basic design requirements for the knee cam.

Quantities (unit)	$U_{0}$	$U_{1}$	$U_{2}$
(x,y) (mm, mm)	(0, 20)	[−24.5sin(12°), 24.5cos(12°)]	[−lsin(45°), lcos(45°)]
(x,y') (mm, 1)	[0, tan(α)]		[−lsin(45°), tan(45°)]

To facilitate the calculation, the data in Table 1 are based on Figure 4, where the origin is the rotation center of the cam, and the X–Y coordinate system conforms to the Cartesian coordinates.

To reduce the machining cost of the cam, the contour equation of the cam is set as a fourth-order polynomial based on five design requirements. The function expression is

$$\{\begin{array}{c} y=ax^{4}+bx^{3}+cx^{2}+dx+e\\ \dot{y}=4ax^{2}+3bx^{2}+2cx+d. \end{array}.$$

In the previous formula, a, b, c, d, and e are unknown quantities that need to be calculated.

Bring (0,20) , (−24.5sin(12°) , 24.5cos(12°)) , (−lsin(45°) , lcos(45°)) , (0,tan(α)) , and (−lsin(45°) , tan(45°)) into the equations and simplify the equations to get the following formula, in which the unit of length used is the millimeter.

$$\{\begin{array}{c} e=20\\ {\left(-24.5sin\left(12{}^{\circ}\right)\right)}^{4}a+{\left(-24.5sin\left(12{}^{\circ}\right)\right)}^{3}b+{\left(-24.5sin\left(12{}^{\circ}\right)\right)}^{2}c+\left(-24.5sin\left(12{}^{\circ}\right)\right)d+e=24.5cos\left(12{}^{\circ}\right)\\ {\left(-lsin\left(45{}^{\circ}\right)\right)}^{4}a+{\left(-lsin\left(45{}^{\circ}\right)\right)}^{3}b+{\left(-lsin\left(45{}^{\circ}\right)\right)}^{2}c+\left(-lsin\left(45{}^{\circ}\right)\right)d+e=lcos\left(45{}^{\circ}\right)\\ d=tan\left(\alpha \right)\\ 4a{\left(-lsin\left(45{}^{\circ}\right)\right)}^{2}+3b{\left(-lsin\left(45{}^{\circ}\right)\right)}^{2}+2c\left(-lsin\left(45{}^{\circ}\right)\right)+d=tan\left(45{}^{\circ}\right). \end{array}.$$

Turn the equations into matrix equations to get

$$\begin{array}{l} \left[\begin{array}{ccc} {\left(-24.5sin\left(12{}^{\circ}\right)\right)}^{4} & {\left(-24.5sin\left(12{}^{\circ}\right)\right)}^{3} & {\left(-24.5sin\left(12{}^{\circ}\right)\right)}^{2}\\ {\left(-lsin\left(45{}^{\circ}\right)\right)}^{4} & {\left(-lsin\left(45{}^{\circ}\right)\right)}^{3} & {\left(-lsin\left(45{}^{\circ}\right)\right)}^{2}\\ 4{\left(-lsin\left(45{}^{\circ}\right)\right)}^{2} & 3{\left(-lsin\left(45{}^{\circ}\right)\right)}^{2} & 2\left(-lsin\left(45{}^{\circ}\right)\right) \end{array}\right]\left[\begin{array}{c} a\\ b\\ c \end{array}\right]+\left[\begin{array}{c} \left(-24.5sin\left(12{}^{\circ}\right)\right)tan\left(\alpha \right)+20\\ \left(-lsin\left(45{}^{\circ}\right)\right)tan\left(\alpha \right)+20\\ tan\left(\alpha \right) \end{array}\right].\\ =\left[\begin{array}{c} 24.5cos\left(12{}^{\circ}\right)\\ lcos\left(45{}^{\circ}\right)\\ tan\left(45{}^{\circ}\right) \end{array}\right]. \end{array}$$

The values of a and b can be obtained as

$$\begin{array}{l} \left[\begin{array}{c} a\\ b\\ c \end{array}\right]=\left[\begin{array}{ccc} \frac{1}{{\left(-24.5sin\left(12{}^{\circ}\right)\right)}^{2}{\left(-24.5sin\left(12{}^{\circ}\right)+lsin\left(45{}^{\circ}\right)\right)}^{2}} & \frac{-2\times 24.5sin\left(12{}^{\circ}\right)+3lsin\left(45{}^{\circ}\right)}{{\left(-lsin\left(45{}^{\circ}\right)\right)}^{3}{\left(-24.5sin\left(12{}^{\circ}\right)+lsin\left(45{}^{\circ}\right)\right)}^{2}} & \frac{-1}{{\left(-lsin\left(45{}^{\circ}\right)\right)}^{2}\left(-24.5sin\left(12{}^{\circ}\right)+lsin\left(45{}^{\circ}\right)\right)}\\ \frac{2lsin\left(45{}^{\circ}\right)}{{\left(-24.5sin\left(12{}^{\circ}\right)\right)}^{2}{\left(-24.5sin\left(12{}^{\circ}\right)+lsin\left(45{}^{\circ}\right)\right)}^{2}} & \frac{-2\times \left({\left(-24.5sin\left(12{}^{\circ}\right)\right)}^{2}-2\times {\left(-lsin\left(45{}^{\circ}\right)\right)}^{2}\right)}{{\left(-lsin\left(45{}^{\circ}\right)\right)}^{3}{\left(-24.5sin\left(12{}^{\circ}\right)+lsin\left(45{}^{\circ}\right)\right)}^{2}} & \frac{\left(-24.5sin\left(12{}^{\circ}\right)-lsin\left(45{}^{\circ}\right)\right)}{{\left(-lsin\left(45{}^{\circ}\right)\right)}^{2}\left(-24.5sin\left(12{}^{\circ}\right)+lsin\left(45{}^{\circ}\right)\right)}\\ \frac{{\left(-lsin\left(45{}^{\circ}\right)\right)}^{2}}{{\left(-24.5sin\left(12{}^{\circ}\right)\right)}^{2}{\left(-24.5sin\left(12{}^{\circ}\right)+lsin\left(45{}^{\circ}\right)\right)}^{2}} & \frac{-\left(-3\times {\left(-24.5sin\left(12{}^{\circ}\right)\right)}^{2}+4\left(-24.5sin\left(12{}^{\circ}\right)\right)\left(-lsin\left(45{}^{\circ}\right)\right)\right)}{{\left(-lsin\left(45{}^{\circ}\right)\right)}^{2}{\left(-24.5sin\left(12{}^{\circ}\right)+lsin\left(45{}^{\circ}\right)\right)}^{2}} & \frac{24.5sin\left(12{}^{\circ}\right)}{\left(-lsin\left(45{}^{\circ}\right)\right)\left(-24.5sin\left(12{}^{\circ}\right)+lsin\left(45{}^{\circ}\right)\right)} \end{array}\right].\\ \times \left[\begin{array}{c} 24.5\cos\left(12{}^{\circ}\right)+24.5\sin\left(12{}^{\circ}\right)tan\left(\alpha \right)-20\\ \frac{l\sqrt{2}+l\sqrt{2}tan\left(\alpha \right)-40}{2}\\ 1-tan\left(\alpha \right) \end{array}\right]. \end{array}$$

Furthermore, based on the known cam equation, the equation of the straight line where the spring force lies can be obtained:

$$Y=\left(-\frac{1}{\dot{y}}\right)\left(X-x\right)+y.$$

Furthermore, the moment arm equation of the elastic force can be obtained as

$$d=\frac{\left|y+\left(\frac{1}{\dot{y}}\right)x\right|}{\sqrt{{\left(\frac{1}{\dot{y}}\right)}^{2}+1}}.$$

After introducing y and $\dot{y}$ , the equation can be simplified to

$$d=\frac{\left|\left(ax^{4}+bx^{3}+cx^{2}+dx+e\right)+\frac{1}{\left(4ax^{3}+3bx^{2}+2cx+d\right)}x\right|}{\sqrt{{\left(\frac{1}{4ax^{3}+3bx^{2}+2cx+d}\right)}^{2}+1}}.$$

After defining α⊂(0,0.45Π)∪(0.6Π,Π) and l⊂(20,40) , the three-dimensional graph of d(α,l) at the contact point can be obtained. Based on Figure 1, l→40,α→0/∏ will cause $d\left(\alpha ,l\right)\to d_{\max}\left(\alpha ,l\right)$ .

To make the exoskeleton fit the motion law of the human body, the monotonicity of the cam radius needs to be restricted. A judgment algorithm based on (α,l) is defined in Matlab:

$$\{\begin{array}{c} L=L\left(x\left(\alpha ,l,i\right),y\left(x\left(\alpha ,l,i\right)\right)\right)=\sqrt{x^{2}\left(\alpha ,l,i\right)+y^{2}\left(\alpha ,l,i\right)};x\subset \left(-l\sin\left(\frac{\Pi }{4}\right)\text{,0}\right),i\subset \left(1,n\right)\\ k\left(\alpha ,l\right)=\frac{\sum_{i=1}^{n}\left|L\left(\alpha ,l,i\right)-fliplr\left(sort\left(L\right)\right)\right|}{n}\\ \{\begin{array}{c} K=1;k\left(\alpha ,l\right)\leq \mu \\ K=1;k\left(\alpha ,l\right)≻\mu \end{array} \end{array}.$$

In the previous formula, L=L(x(α,l,i),y(x(α,l,i))) is the distance from a point on the cam profile to its center of rotation, the function fliplr(sort(L)) is to arrange the matrix in order from large to small, and the function of the threshold μ is to fully accommodate the influence of calculation errors on the results.

**FIGURE 5:**
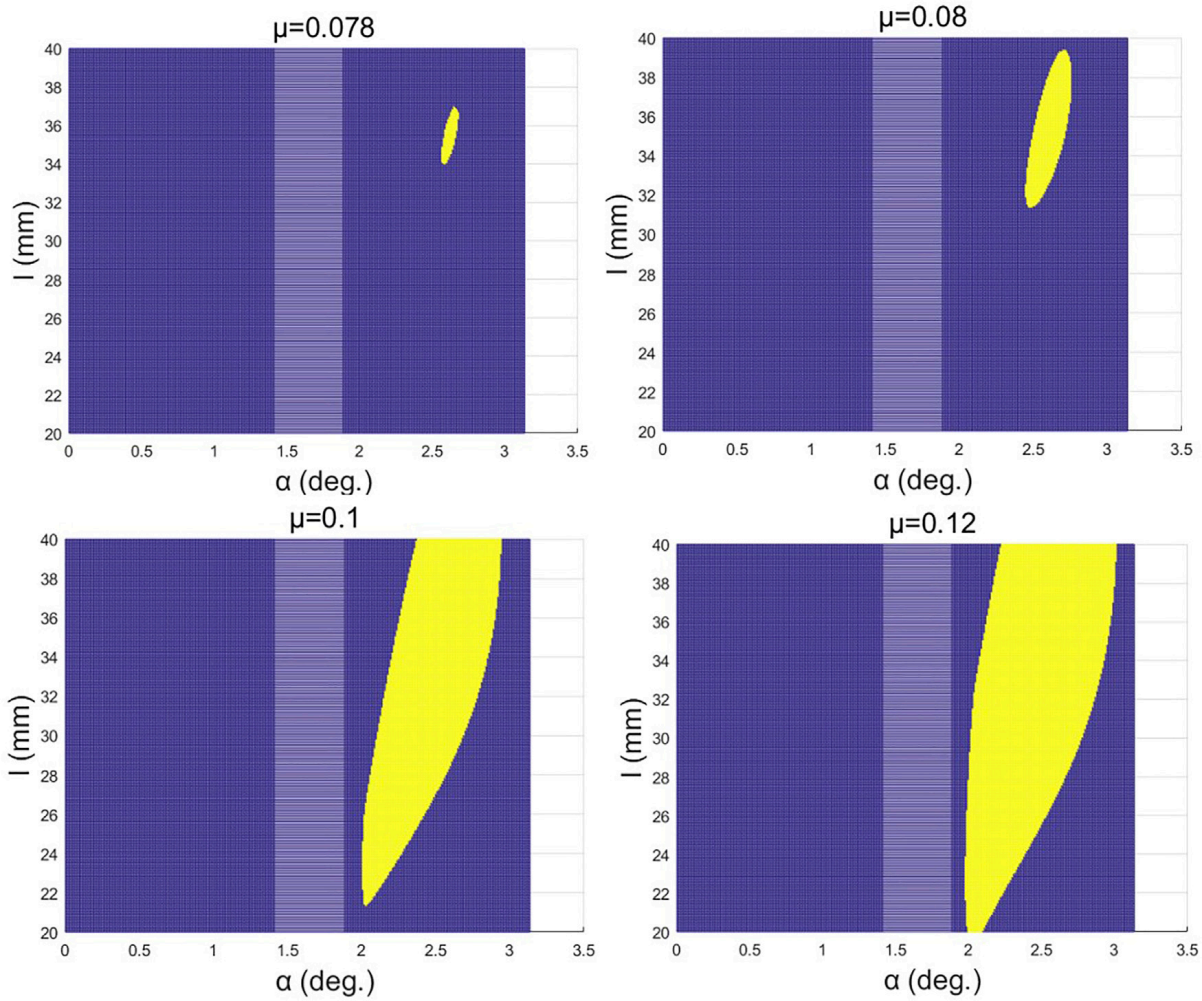
Monotonicity of the cam profile equation.

The three-dimensional graph of K=K(α,l) with μ=0.08 , μ=0.1 , μ=0.12 , and μ=0.15 is obtained, respectively (Figure 5).

**FIGURE 6:**
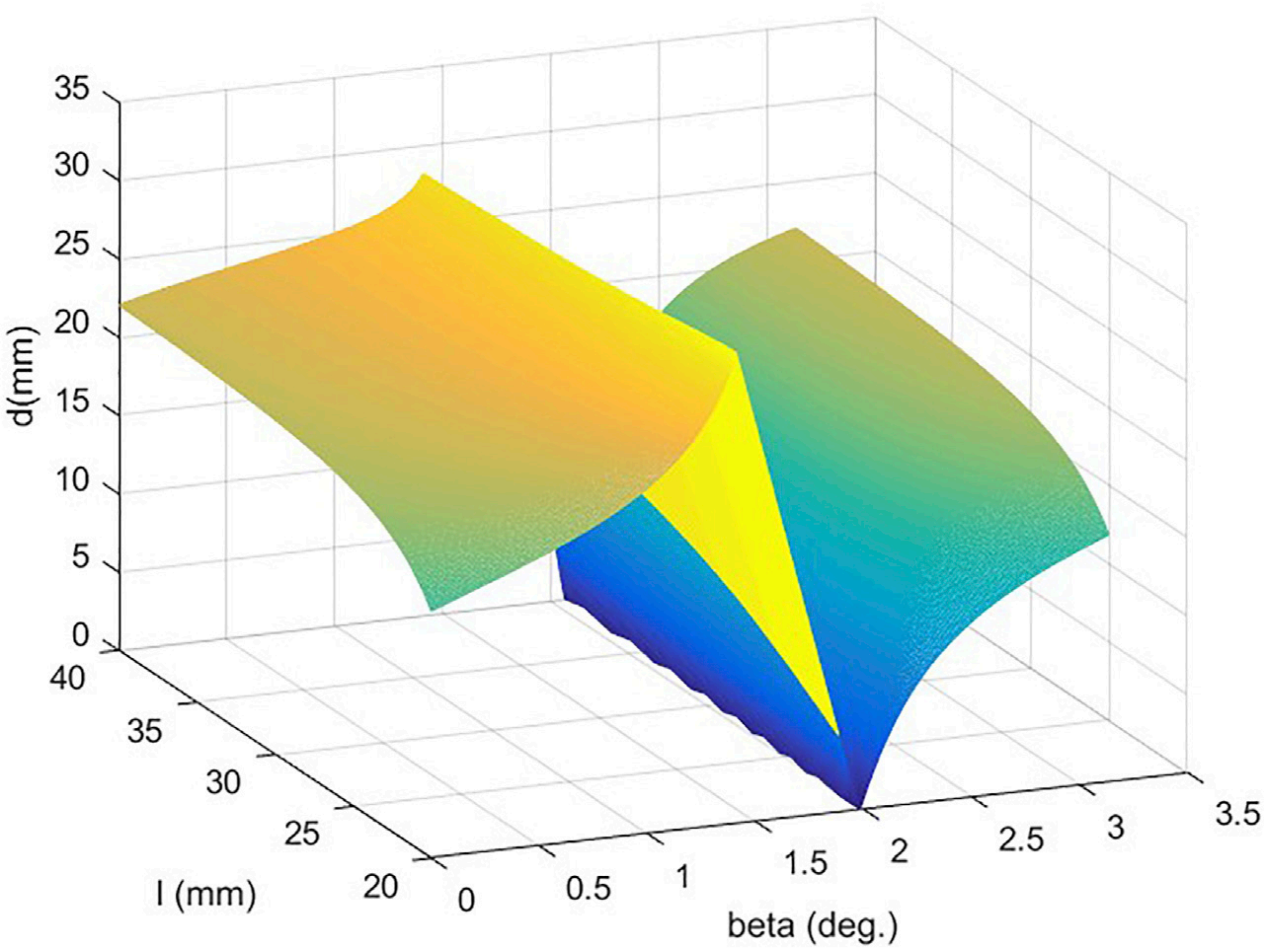
Moment arm at the contact point.

In Figure 5, the yellow area represents K=1 and the blue area represents K=0 . Based on Figure 6, to get the maximum moment arm, the upper right boundary of the yellow area needs to be considered. To obtain high-precision design requirements, this article selects μ=0.078 ; at this time, $d_{\max}\left(\alpha ,l\right)=19.3$ , l=36.5 , and α=2.675 .

## 4 The Parameter Design of the Elastic Component in the Coupling Mechanism

The coupling mechanism is to transfer or convert the motion form of one joint into the clutch signal for the other joint, and for the passive lower extremity exoskeleton, the clutch signal is the decisive factor for establishing a connection between the energy storage element and the joint movement and, according to the work needs of the lower limb exoskeleton, should be generated and transmitted in time, which is the sensitivity requirement. In this article, the contact between the pawl and the roller follower is the decisive factor for the effectiveness of the coupling mechanism, and the parameter design of the knee torsion spring, especially the initial moment of the torsion spring, which exerts the driving force to the roller follower will be the key to the design of the coupling mechanism.

### 4.1 The Parameter Design Criteria for the Knee Torsion Spring

To achieve the power assistance of the exoskeleton, in addition to the sensitivity requirements, the parameter design also needs to meet another requirement: the torque that can be provided by the knee torsion spring can not only meet the movement needs of the exoskeleton calf, and only by providing a higher driving torque, can excess mechanical energy be transmitted to the human body.

Based on the following formula, in the process of the power assistance, the torque provided by the knee torsion spring will linearly decrease with the reduction of the rotation angle of the human knee joint. To ensure that the knee torsion spring can continuously provide positive power assistance to the human body, when the initial torque of the torsion spring and the driving torque required for the exoskeleton’s calf swing are clarified, the slope of the torsion spring needs to be selected. Its essence is to make requirements for the stiffness coefficient of the knee torsion.

$$T=K\times \theta +T_{0}.$$

In the previous formula, K , T , θ , and $T_{0}$ , respectively, represent the stiffness, torque, moment angle, and initial torque of the knee torsion spring.

**FIGURE 7:**
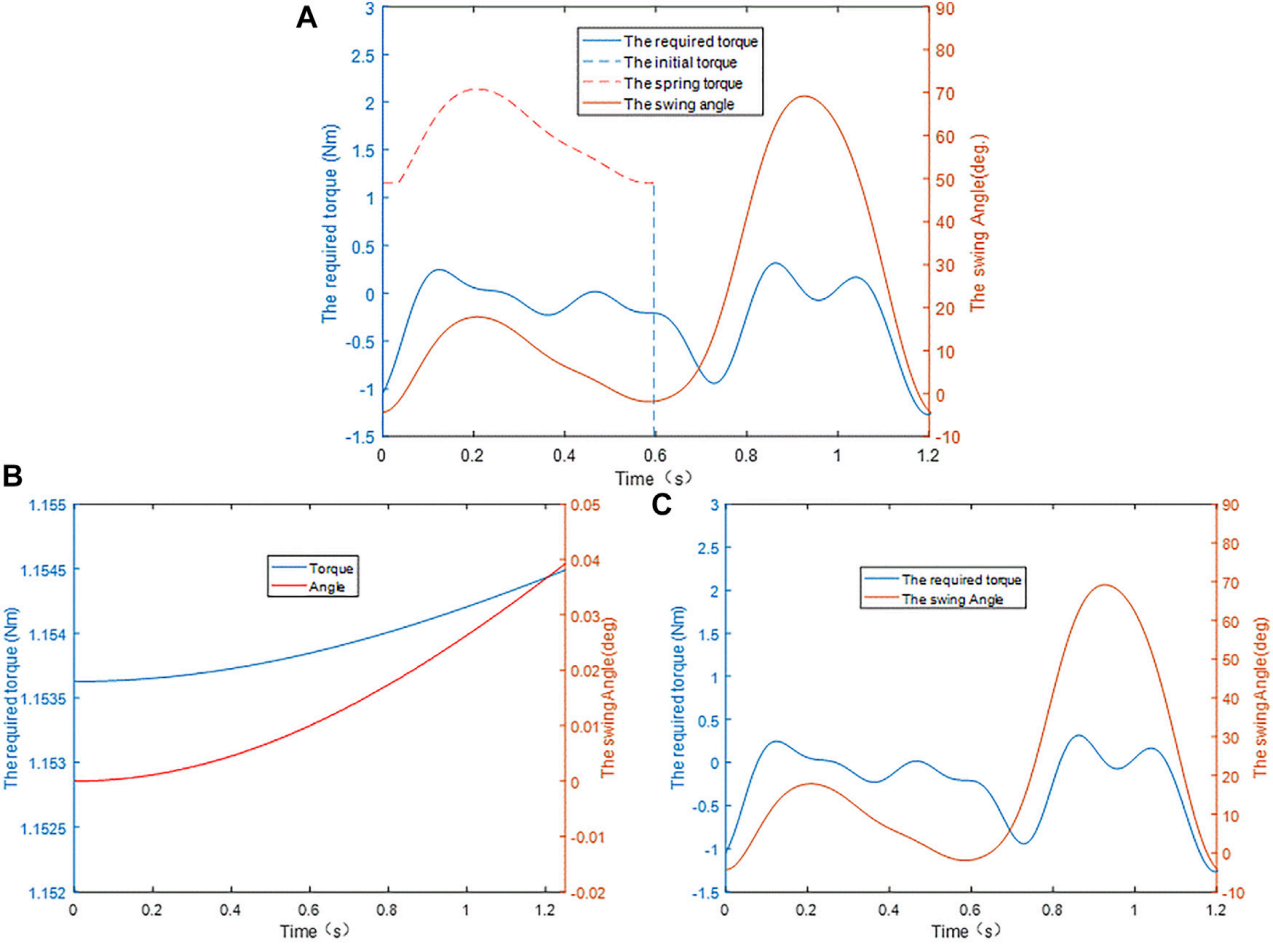
**(A)** Parameter design of the knee torsion spring. **(B)** Driving torque of the cam. **(C)** Driving torque of the exoskeleton calf.

The aforementioned torque curves are shown in Figure 7A. Based on the geometric law of each curve, the requirement for the knee torsion spring is to ensure that during the support period, its torque curve ( $Y_{1}$ ) is always higher than the torque curve required by the exoskeleton calf ( $Y_{2}$ ), where the ratio of the derivative of $Y_{1}$ to $Y_{2}$ is the stiffness value of the torsional spring of knee joint.

$$k=\frac{{\dot{Y}}_{1}}{{\dot{Y}}_{2}}.$$

### 4.2 The Initial Force of the Knee Torsion Spring

The CAD model of the exoskeleton constructed in SolidWorks was imported into Adams for dynamic and kinematic simulations. To overcome the influence of gravity in the initial state on the basis of ensuring the uniqueness of the variable, this article imposes a small angular velocity on the cam. The principle is that to maintain a constant angular velocity of the cam, the system needs to apply a driving torque to it. To make the measured joint torque meet the accuracy requirements, the applied speed-driven equation is defined as −0.001∗sin (time) (the minus sign in the equation is related to the direction of rotation of the cam).

The simulation results are shown in Figure 7B. In the entire simulation interval, the torque range applied by the knee torsion spring to the rotary joint of the cam is [1.1525, 1.1540], and the output torque at the zero time is selected to be 1.15 Nm, which can ensure that there are two significant digits.

### 4.3 The Stiffness of the Knee Torsion Spring

Because the exoskeleton calf under different swing states has different requirements for its driving torque, to ensure that the exoskeleton can continuously provide positive work for the human body during the entire working period, it is necessary to measure the change of the driving torque. Because the exoskeleton calf is tied to the human calf, the two have the same motion state, so it is necessary to apply the motion data of the human knee joint to the exoskeleton knee joint and measure the driving torque it receives during the entire gait cycle. The simulation result is shown in Figure 7C, where the available range of the driving torque is between [0, 0.595], and the position where the extreme value of the torque appears in this interval is (0.125, 0.2481).

Based on the linear correlation principle between the torque of the knee torsion spring and the motion angle of the knee joint, $Y_{1}$ under the minimum condition can be obtained after the unit transformation and proportional scaling of $Y_{2}$ . At this time, the proportional value of the scaling represents the minimum stiffness that the knee torsion spring should have. Therefore, $Y_{1}$ with the lower stiffness should meet two requirements: one is the linear relationship with $Y_{2}$ and the other is it should be over the point (0.595, 1.1538) and the point (0.125, 0.248), respectively.

Based on Figure 7C, the preload of 1.1538 Nm is greater than the maximum torque of 0.2481 Nm required by the exoskeleton calf. It can be judged from the geometric position of each curve in Figure 1 that the characteristic curve that meets the requirements can be obtained only by setting the stiffness of the knee torsion spring to a negative value. However, the negative stiffness of the knee torsion spring is not desirable, which is contrary to the original design intention of the knee energy storage mechanism in this article. However, it is not difficult to find from another perspective that when the positive stiffness parameters of any value are adopted, the torque of the torsional spring will always be higher than the driving torque required when the leg swings. The essential significance is that not all mechanisms need to set the lower limit on the spring stiffness, because the calculation of the lower limit is affected by the size, shape, and weight of the mechanism. However, the further selection of the torsional spring stiffness should first select the appropriate load moment on the basis of human comfort, and then scale the angle change of the human knee joint. This process is similar to the calculation of the lower limit value of the torsional spring stiffness.

## 5 The Result Discussion and Index Design for the Exoskeleton Effectiveness

Considering that the simulation of the model can directly reflect the change of the joint torque, it can avoid the influence caused by the non-unique variable (van den Bogert, 2003; van Dijk et al., 2011; Wiggin et al., 2011; van Dijk and Van der Kooij, 2014). To verify the walking assistance effect of the lower limb exoskeleton, the coupling body consists of the human body model, and the exoskeleton established in SolidWorks is imported into Adams, and applied motion data at normal walking speed to the lower limb joints to get the efficiency curves of the hip and knee joints when the exoskeleton is working and not, respectively, and then by comparing the average values of the absolute value of the joint power to comprehensively judge the performance of the exoskeleton (van Dijk et al., 2011; Wiggin et al., 2011).

### 5.1 The Functional Verification of the HESM

**FIGURE 8:**
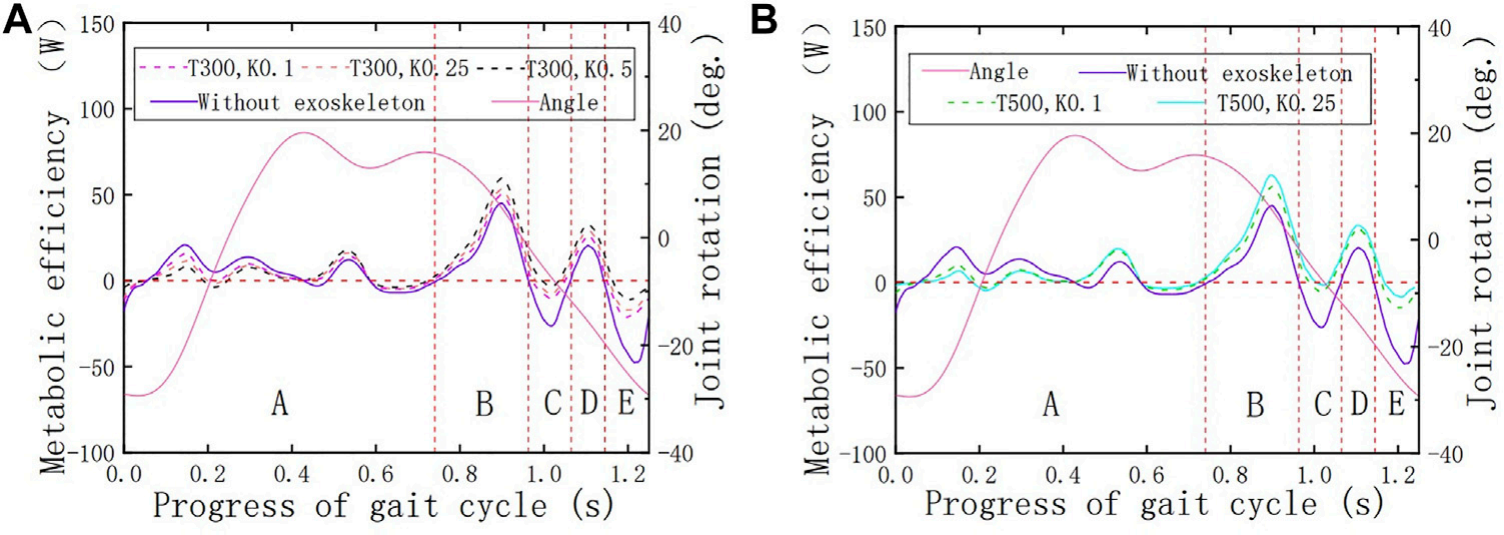
**(A)** Verification of the HESM under the initial force of 300 N. **(B)** Verification of the HESM under the initial force of 500 N.

**FIGURE 9:**
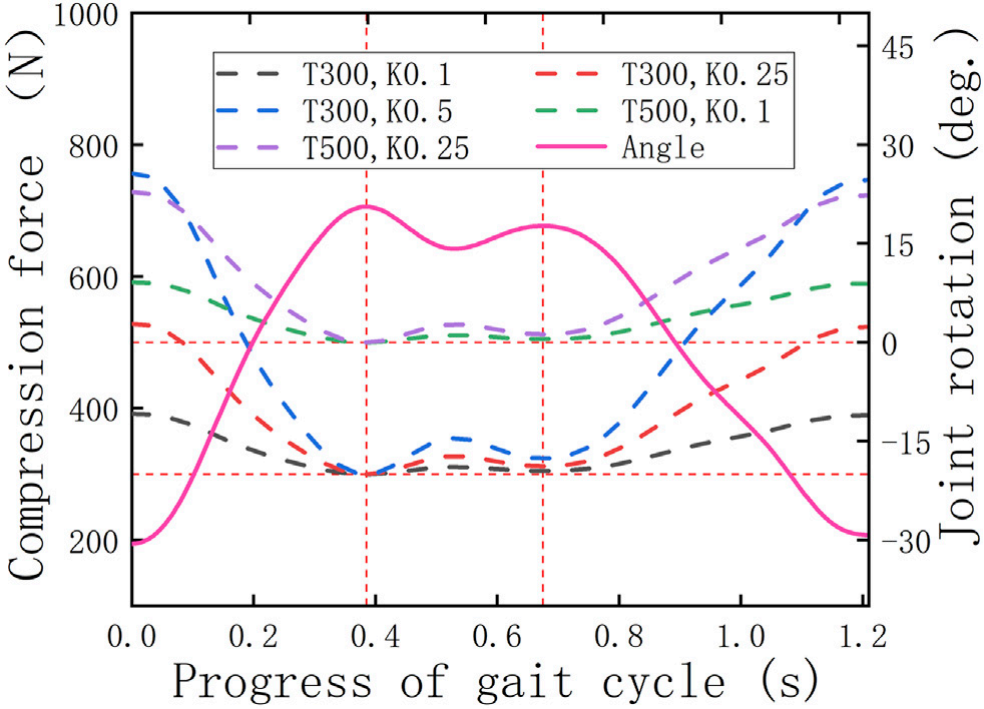
Tension changes of the hip spring.

The hip joint powers in the two working conditions are shown in Figures 8A,B. To test the influence of the initial torque and the spring stiffness on the HESM, several different parameter collocations were selected (Figures 8A,B) (Wiggin et al., 2011; Yihua et al., 2019), where T represents the initial force (N) and K represents the spring stiffness (N/m):

Figure 9 contains the angle curve of the hip joint and the tension curves of the hip straight spring. First of all, it is worth affirming that the tension changes follow the angle change, which shows that the parameter settings of the motion pair, collision force, and spring force are correct, and further shows that the HESM completes the energy storage and release in time. When the tension of the spring increases, it indicates that the human body inputs energy to the energy storage mechanism, and on the contrary, it represents the energy storage mechanism, which outputs the stored energy to the human body.

Based on Figures 8A,B, it is worth noting that on the whole, the power curves of the energy metabolism when the exoskeleton is working are closer to the zero-scale line than when the exoskeleton is not working, which fundamentally indicates that the exoskeleton designed in this article successfully follows the previous design idea and is a feasible design scheme. Second, based on the variation tendency of the simulation results with the exoskeleton with different parameters, the larger the initial torque and spring stiffness, the greater impact it brings on the hip energy consumption. Specifically, the decrease of joint power in the front interval is due to the forward swing of the hip joint, which is the present working area of the HESM to assist the body motion. This uses the joint negative work stored by the HESM to compensate for the joint positive work. At the same time, it cannot be ignored that the power curves in the latter part are higher than when the exoskeleton is not working, which is a treatment method for the instability characteristics of the transformation node between the positive and negative works.

Two things in the simulation results were beyond the expectations. First, the simulation results of the man–machine coupling model all have a negative value region in the vicinity of 0.2 s, which will directly weaken the assistance effect of the exoskeleton on the hip joint. The immediate reason is that the parameter combination selected in this article exceeds the endurance of the hip joint (Collins et al., 2015; Lee and Wang, 2015; Alamdari et al., 2019). Second, there is an area with a positive power value near 1.1 s, which is abnormal to the related data. Although it does not waste the total energy of the human body, a wide range of contrary signs of the value of number will seriously affect the auxiliary effect of the lower extremity exoskeleton (van den Bogert, 2003; van Dijk et al., 2011; Wiggin et al., 2011; van Dijk and Van der Kooij, 2014; Collins et al., 2015; Lee and Wang, 2015; Yandell et al., 2019).

**FIGURE 10:**
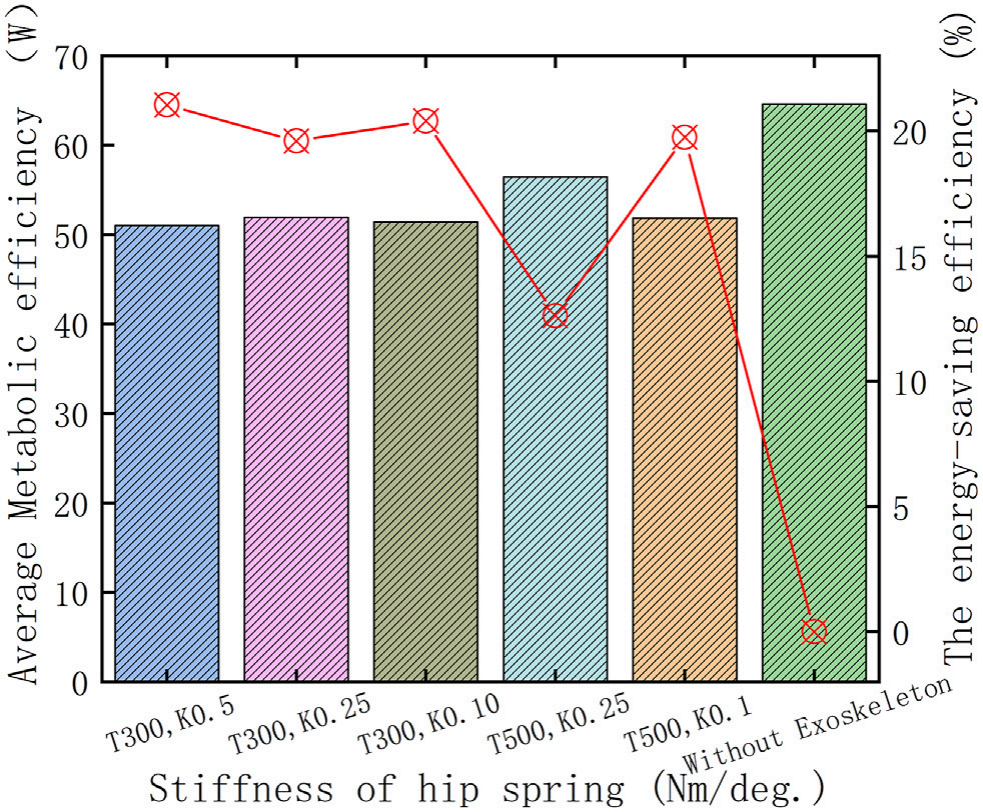
Energy-saving efficiency of the HESM fort the hip joint.

To verify the walking assistance ability of the HESM under different parameter combinations at an objective level, this article discusses the energy-saving efficiencies from the perspective of the average value of the absolute value of the joint power, and the results are shown in Figure 10. The reason is the power curves of the hip joint are composed of a series of discrete points. Based on the calculation method of the ultimate walking assistance efficiency of the HESM shown in the following formula, the result in this article is 52.36%, which is theoretically much higher than that of other exoskeleton designs (van den Bogert, 2003; Banala et al., 2006; Rahman et al., 2007; van Dijk et al., 2011; Wiggin et al., 2011; van Dijk and Van der Kooij, 2014; Collins et al., 2015; Lee and Wang, 2015; Hung et al., 2017; Alamdari et al., 2019; Lovrenovic and Doumit, 2019; Yandell et al., 2019; Zhou et al., 2020), and the existing simulation results are much smaller than it. The reason for these huge differences includes not only the two unexpected situations mentioned before but also a great difficulty in the transformation degree and identification cycle between the joint positive and negative works.

$$\psi =100\%-\frac{\left|\frac{\sum_{i=1}^{N_{A}}w_{i}}{N_{A}}+\frac{\sum_{j=1}^{N_{C}}w_{j}}{N_{C}}+\frac{\sum_{k=1}^{N_{E}}w_{k}}{N_{E}}\right|+\left|\frac{\sum_{l=1}^{N_{B}}w_{l}}{N_{B}}\right|+\left|\frac{\sum_{m=1}^{N_{D}}w_{m}}{N_{d}}\right|}{\frac{\sum_{i=1}^{N_{A}}\left|w_{i}\right|}{N_{A}}+\frac{\sum_{l=1}^{N_{B}}w_{l}}{N_{B}}+\frac{\sum_{j=1}^{N_{C}}w_{j}}{N_{C}}+\frac{\sum_{m=1}^{N_{D}}w_{m}}{N_{D}}+\frac{\sum_{k=1}^{N_{E}}w_{k}}{N_{E}}}\times 100\%\;\;N_{A}+N_{B}+N_{C}+N_{D}=N_{TN}.$$

In the previous formula, $w_{i}$ , $w_{l}$ , $w_{j}$ , $w_{m}$ , and $w_{k}$ , respectively, represent the joint power of the discrete points without exoskeleton in the A, B, C, D, and E intervals, and $N_{TN}$ represents the total number of scattered points during the gait cycle (Figure 8).

### 5.2 The Functional Verification of the KESM

**FIGURE 11:**
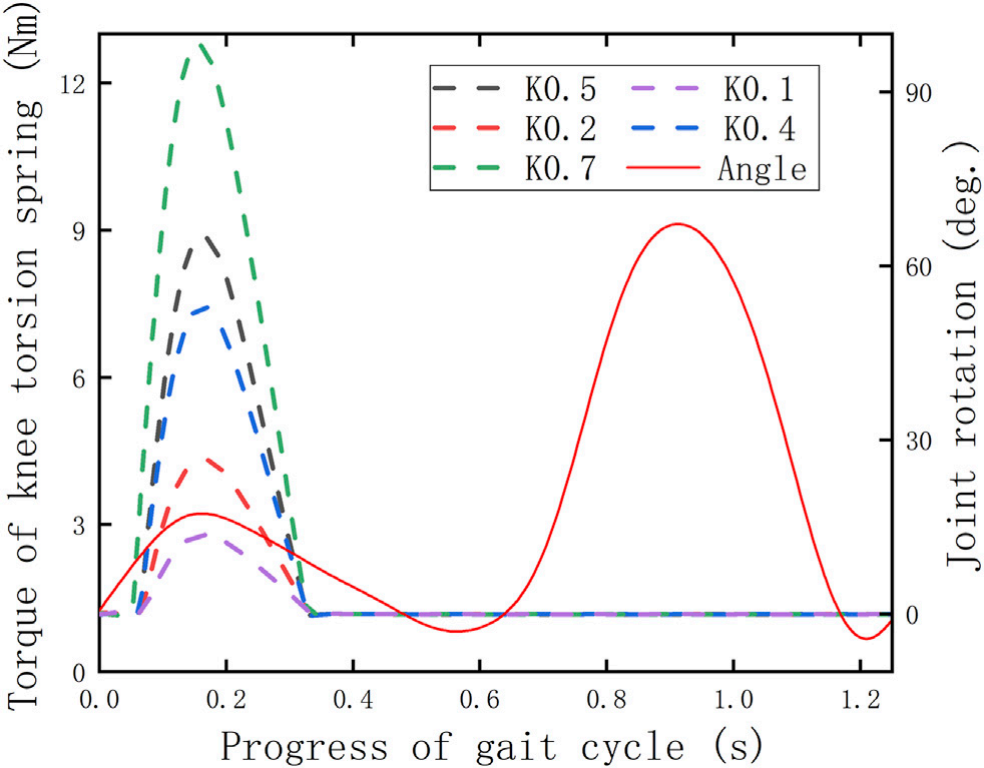
Torque changes of the knee torsion spring.

To preliminarily verify whether the KESM works according to the predetermined design criteria, the torque curves of the knee torsion spring are derived in the post-processing interface after simulating the human–machine coupling model, and the change is shown in Figure 11, where T represents the initial moment (Nm) and K represents the spring stiffness (Nm/deg.). It needs to be stated in advance that the initial torque of the torsional spring of the knee joint is set as 1.15 Nm, which ensures it can overcome the gravity of the roller follower at zero time.

**FIGURE 12:**
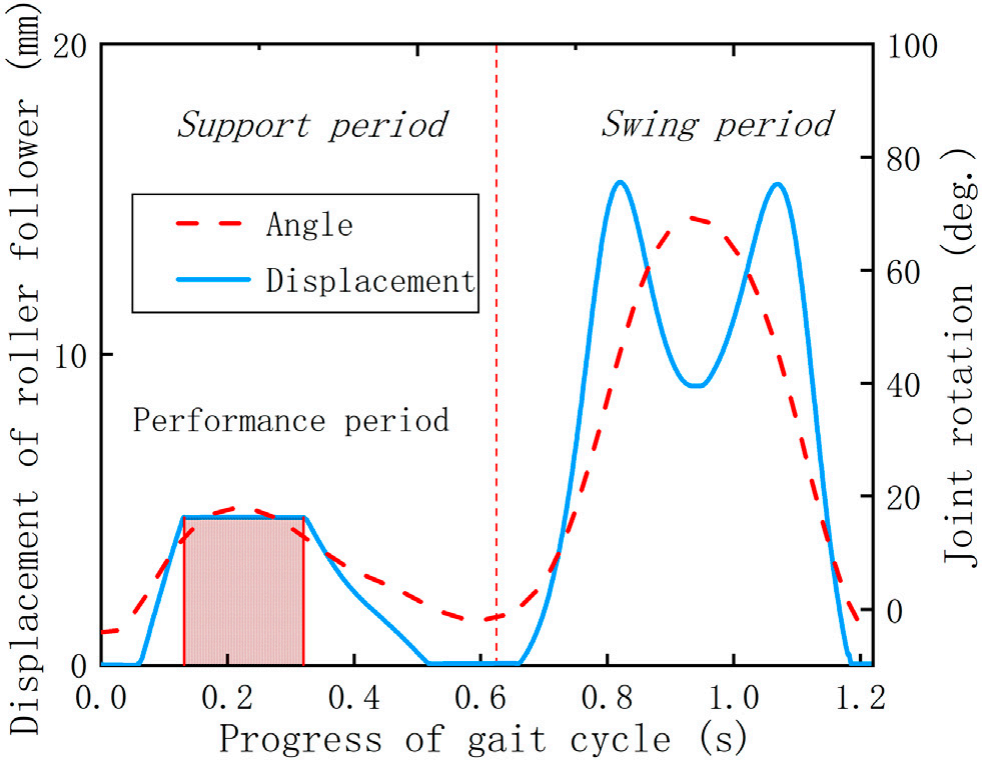
Motion characteristics of the coupling mechanism.

Based on the simulation results (Figure 11), it can be cleared that except for one peak, all the other torque curves under different parameters present an approximately horizontal state in the whole gait cycle, where the time range is consistent with the cutoff period of the displacement curve of the roller follower (Figure 12). The reason is that it proves that the knee torsion spring only plays a role in energy storage in the stance period, and there is no obvious energy delivery with the human body in the swing period.

**FIGURE 13:**
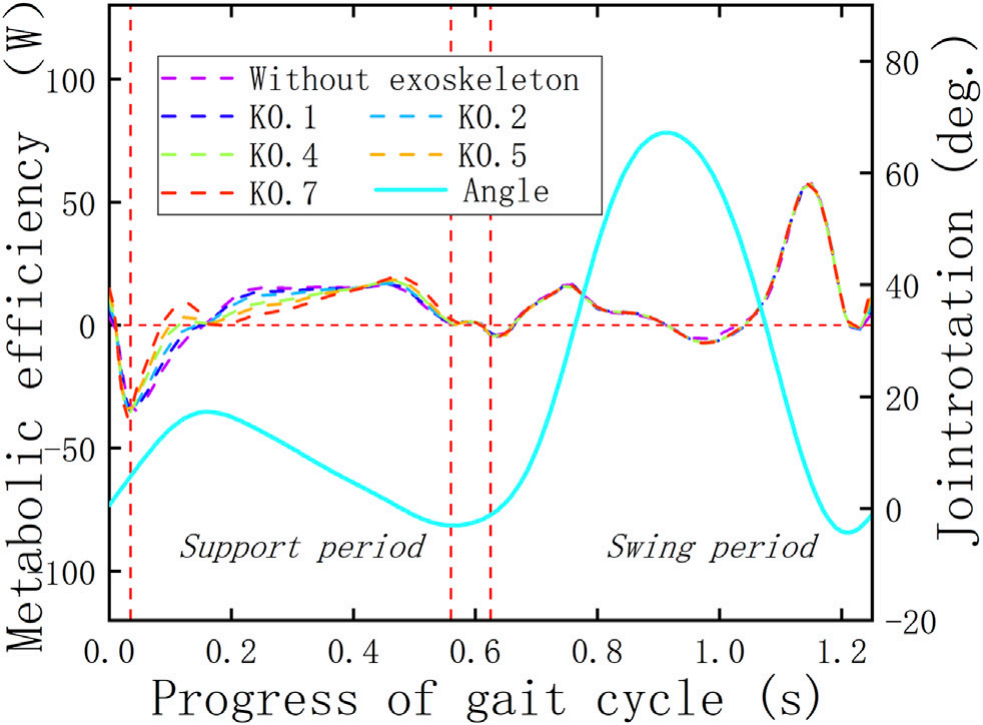
Verification of the HESM.

To determine whether the KESM is of positive significance, it is necessary to combine the displacement curve of the roller follower and the angle curve of the knee joint to make a combined analysis with the knee joint powers with and without the exoskeleton. The previous curves are shown in Figures 11–13. In the whole gait cycle, these power curves under different working conditions show approximately the same state except for some differences in the stance period. The time range when the differences appear is consistent with the interaction time between the roller follower and the pawl, indicating that the KESM has no significant influence on the joint power in the swing period and completes the energy transfer between humans and machines in the stance period.

The verification criterion of the KESM is similar to that of the HESM because when the exoskeleton is working, the dispersion of the joint power curve relative to the zero-scale line is smaller than that when the exoskeleton is not working, which can prove that the design of the KESM has a successful foundation. In fact, by comparing the joint power curves under the two working conditions (Wiggin et al., 2011; Alamdari et al., 2019), it can be seen that when the knee joint is bent, the KESM stores the negative work, in which direction is opposite to the knee movement and compensate the positive work required for the positive movement of the knee joint when the knee joint extends straightforward.

**FIGURE 14:**
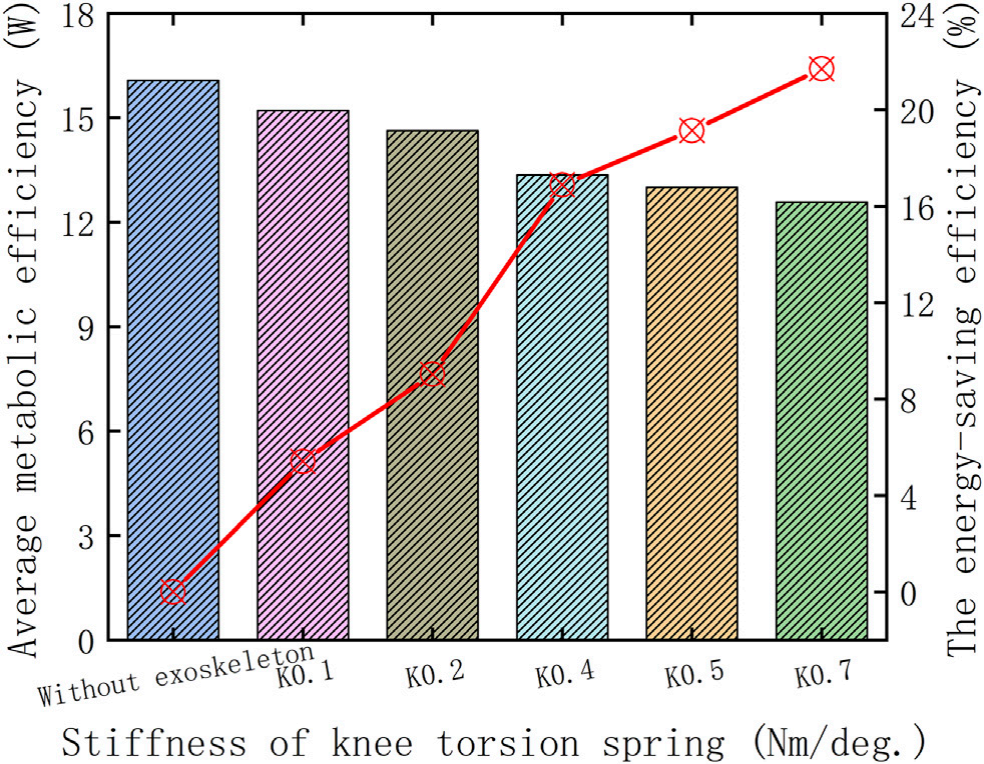
Walking assistance ability of the KESM in the stance period.

In this article, the arithmetic mean of the absolute value is calculated for several discrete points of the power curves of joint energy metabolism, and the results are shown in Figure 14. Based on the calculation method, the ultimate walking assistance efficiency of the KESM is as follows: the result in this article is 66.72%, which is theoretically much higher than that of other exoskeleton designs (van den Bogert, 2003; Banala et al., 2006; Rahman et al., 2007; van Dijk et al., 2011; Wiggin et al., 2011; van Dijk and Van der Kooij, 2014; Collins et al., 2015; Lee and Wang, 2015; Hung et al., 2017; Alamdari et al., 2019; Lovrenovic and Doumit, 2019; Yandell et al., 2019; Zhou et al., 2020), and the simulation result is still less than the theoretical value. The extreme value of the principle of mutual offset between joint positive and joint negative work in the whole gait range will be the focus for future improvement.

$$\psi =100\%-\frac{\frac{\sum_{o=1}^{N_{F}}\left|w_{o}\right|}{N_{F}}+\frac{\sum_{p=1}^{N_{G}}\left|w_{p}\right|}{N_{G}}+\frac{\sum_{q=1}^{N_{H}}\left|w_{q}\right|}{N_{H}}}{\frac{\sum_{r=1}^{N_{SP}}\left|w_{r}\right|}{N_{SP}}}\times 100\%\;N_{F}+N_{G}+N_{H}=N_{SP},$$

where $N_{SP}$ represents the scattered points of the without exoskeleton curve during the stance period (Figure 8).

## 6 Conclusion

Aging brings inconvenience and confusion to the daily activities of the elderly. To alleviate such problems from the direction of mechanical engineering, this article designed a hip–knee coupling exoskeleton with offset theory to provide people with the walking assistance function, in which the offset theory was discussed in detail, and based on it, an innovative hip–knee coupling theory was proposed, which includes the motion planning, system modeling, optimization algorithms, and sensitive conditions. The main content of the hip–knee coupling was to extract the motion law of the hip joint and convert it into the clutch control signal of the KESM. Specifically, the structural design of the HESM was to convert the stroke of the cam mechanism into the deformation of the spring to realize the energy transfer between the human and the machine. The structural design of the KESM was to not produce any form of interference, including movement and geometry, to the human knee joint during the swing period, and realize the power assistance function during the stance period.

To achieve the exoskeleton design successfully, the simplified mathematical model of the coupling mechanism was established to optimize the load environment of the exoskeleton system, and on that basis, an algorithm was proposed to determine the monotonicity of the cam profiles. In addition, the sensitivity condition was proposed for the coupling mechanism, and on that basis, the human–machine interaction model for the KESM was established to select the elastic parameters of the coupling mechanism. To verify the effectiveness of the exoskeleton, based on the offset theory, a design index for the energy-saving efficiencies of the exoskeleton was proposed; by monitoring the energy storage components, the energy transfer between humans and machines was preliminarily verified, and by comparing the joint energy consumption power under two working conditions, the effectiveness of the mechanism was finally verified. For the energy storage element, a larger initial force or initial moment and a larger elastic stiffness will have a greater impact on the joint moment of the human body.

Based on the current simulation results, the ultimate walking assistance efficiency can be achieved between 52.36 and 66.72% for the HESM and KESM, respectively, which is theoretically much higher than that of other exoskeleton designs, but the existing exoskeleton prototype did not reach the ideal power assistance effect. This was related to the working cycle and parameter settings of the energy storage element. In addition to the more detailed partition of the working cycle of the lower limb exoskeleton, if the elasticity and stiffness of the components are properly increased, the working capacity of the mechanism will be improved, but this should take into account the comfort of the human body and the exoskeleton effect on the joint moments. Setting the ideal stiffness of the elastic element is a complicated engineering problem, which requires further research and exploration based on this article.

## Data Availability

The raw data supporting the conclusion of this article will be made available by the authors, without undue reservation.
